# High-Throughput *in situ* Root Image Segmentation Based on the Improved DeepLabv3+ Method

**DOI:** 10.3389/fpls.2020.576791

**Published:** 2020-10-19

**Authors:** Chen Shen, Liantao Liu, Lingxiao Zhu, Jia Kang, Nan Wang, Limin Shao

**Affiliations:** ^1^State Key Laboratory of North China Crop Improvement and Regulation, College of Mechanical and Electrical Engineering, Hebei Agricultural University, Baoding, China; ^2^State Key Laboratory of North China Crop Improvement and Regulation, Key Laboratory of Crop Growth Regulation of Hebei Province, College of Agronomy, Hebei Agricultural University, Baoding, China

**Keywords:** root systems, rhizotrons, convolutional neural network, image segmentation, deep-learning

## Abstract

The Rhizotrons method is an important means of detecting dynamic growth and development phenotypes of plant roots. However, the segmentation of root images is a critical obstacle restricting further development of this method. At present, researchers mostly use direct manual drawings or software-assisted manual drawings to segment root systems for analysis. Root systems can be segmented from root images obtained by the Rhizotrons method, and then, root system lengths and diameters can be obtained with software. This type of image segmentation method is extremely inefficient and very prone to human error. Here, we investigate the effectiveness of an automated image segmentation method based on the DeepLabv3+ convolutional neural network (CNN) architecture to streamline such measurements. We have improved the upsampling portion of the DeepLabv3+ network and validated it using *in situ* images of cotton roots obtained with a micro root window root system monitoring system. Segmentation performance of the proposed method utilizing WinRHIZO Tron MF analysis was assessed using these images. After 80 epochs of training, the final verification set F1-score, recall, and precision were 0.9773, 0.9847, and 0.9702, respectively. The Spearman rank correlation between the manually obtained Rhizotrons manual segmentation root length and automated root length was 0.9667 (*p* < 10^–8^), with *r*^2^ = 0.9449. Based on the comparison of our segmentation results with those of traditional manual and U-net segmentation methods, this novel method can more accurately segment root systems in complex soil environments. Thus, using the improved DeepLabv3+ to segment root systems based on micro-root images is an effective method for accurately and quickly segmenting root systems in a homogeneous soil environment and has clear advantages over traditional manual segmentation.

## Introduction

The growth environment of plant roots within the soil is extremely complex. However, soil is a non-transparent medium, so it is difficult to quickly and accurately obtain phenotypic information, which is a critical obstacle to research on root development. Traditional root phenotype research methods mostly use the root drilling and soil column methods as well as other excavation methods (Joslin and Wolfe, 1999; Wasson et al., 2016), followed by washing, screening, dyeing, scanning, and other necessary steps. Accordingly, these destructive sampling methods do not enable phenotypic observations of dynamic root systems *in situ*. To address this limitation, non-destructive observation methods such as X-ray computed tomography (CT) (Mairhofer et al., 2012, 2013; Mooney et al., 2012), nuclear magnetic resonance (NMR) imaging technology (Pflugfelder et al., 2017), laser scanning (Fang et al., 2009), and 3D imaging (Iyer-Pascuzzi et al., 2010; Clark et al., 2011; Topp et al., 2013) have been applied. Although these methods can obtain *in situ* non-destructive images of roots by adjusting and combining different imaging parameters, they cannot enable the observation of larger plants, owing to expense and technical difficulty. The Minirhizotrons method is a relatively balanced method in terms of cost, throughput, and accuracy, and it has the advantages of causing little degradation, enabling *in situ* dynamic visualization, accurate root positioning, and digitization (Liao and Liu, 2008). This method receives more and more information by acquiring *in situ* root images and observing the dynamics of changes in the *in situ* root phenotype in order to systematically study the birth, growth, death, and decomposition processes of roots (Kage et al., 2004; Vamerali et al., 2012). However, the quality of *in situ* image segmentation underlies the quality of the root phenotype results.

Traditionally, root segmentation is performed manually or by semi-automatic interactive segmentation. Manual segmentation relies on researchers visually inspecting all images to identify each root within the intricate soil background, resulting in very low segmentation efficiency, with the additional problem of visual fatigue, which can cause substantial segmentation errors (Abramoff et al., 2004; Le Bot et al., 2010). To improve the efficiency of root segmentation, semi-automatic segmentation combines an automated segmentation algorithm with guidance through human–computer interactions. In this approach, researchers assist auxiliary software in image segmentation based on their own visual observations. For example, GT-Roots requires researchers to specify the segmentation area and selection method (Borianne et al., 2018). Split or WinRHIZO Tron MF requires users to draw a perimeter around a root system in an image with a cursor and manually adjust the perimeter to the diameter of the root system in the picture. Over the course of clicking, the root system is detected by the algorithm and automatically generated, and finally, root segmentation is completed (Lobet et al., 2013; Cai et al., 2015). However, these methods rely on the subjective ability of the personnel to distinguish root systems, introducing the element of visual fatigue, the accompanying proneness to errors, and the inherent difficulty in segmenting large-scale *in situ* root images.

Although Minirhizotrons can help researchers obtain high-definition root images from complex soils, the opacity of soil particles usually poses a challenge for further automation of segmenting root morphology. Traditional image processing methods, such as support vector machine (SVM) (Wilf et al., 2016), and random forest techniques (Breiman, 2001), have improved crop root detection (Singh et al., 2016), were adopted in computer vision. However, some operators and thresholds set by traditional computer vision methods, such as edge detection, morphological filtering, and region growing algorithms, can only segment specific objects and backgrounds and are not practical for all situations. When there are many root coefficients and the background is complex, such artificial target features cannot provide valuable information for subsequent feature learning. Under these conditions, it is difficult to achieve the segmentation accuracy necessary for a fully automated system. With the development of computer vision imaging technology and analytical algorithms, many researches on crop root phenotype data have been deepened in recent years. For example, Falk et al. (2020) proposed a plant root segmentation method based on a computer vision imaging platform and ML and provided biologically relevant time series data on root growth and development for plant breeding applications. González et al. (2020) developed MyROOT 2.0, which uses an automatic and efficient algorithm to detect the root regions of images; this improved the previous version MyROOT, which required manual intervention by the user to define the root area pattern (Betegón-Putze et al., 2019), and also improved the efficiency of batch root detection. Colmer et al. (2020) proposed the SeedGerm system, which integrates automatic seed imaging and machine learning-based phenotype analysis, thus providing a wide range of applications for large-scale phenotype analysis and detection of plant seeds.

Convolutional neural networks (CNNs) are an effective method for replacing traditionally tedious manual target feature extraction. It combines deep learning and computer vision technology to directly extract target features from an input image (LeCun et al., 2015), creating a rich feature hierarchy that can be used for classification without any prior knowledge or cumbersome artificial feature design.

For example, the encoder–decoder-based CNN system RootNav 2.0 (Yasrab et al., 2019) for root image analysis replaces the previous manual and semi-automatic feature extraction system with a very deep multi-task CNN architecture RootNav (Pound et al., 2013). RootNav 2.0 can extract accurate root structures without user interaction, and its speed is increased by 10 times relative to its predecessor. Ruiz-Munoz et al. (2020) designed a framework for the application of deep learning-based SR models to enhance plant root images and demonstrated that the SR model based on deep learning is better than basic bicubic interpolation. AirSurf-Lettuce combines computer vision algorithms and deep learning classifiers to automatically measure the distribution of field iceberg lettuce using super-scale NDVI aerial images, and it has been used to demonstrate the high value of this method in field crop segmentation (Bauer et al., 2019). Wang et al. (2019) proposed a fully automatic root feature extraction method based on CNN called SegRoot and validated its segmentation performance on soybean root images using transfer learning.

Semantic segmentation comprises an important branch of CNNs used for image segmentation, and it is used to measure and segment complex target features on a finer scale. The first application of pixel-level semantic segmentation tasks is the fully convolutional network (FCN) approach (Long et al., 2014), which uses an encoder-decoder structure to automatically extract target features and classify all pixels in an image one by one. In research on root image segmentation based on deep learning, Smith et al. (2020) proposed a U-Net-based root segmentation system; this proposed network architecture is also composed of an encoder–decoder structure. Compared with the traditional machine learning method using Frangi vessel enhancement filter (Frangi et al., 1998), U-net can segment the root morphology in soil images with higher accuracy. However, the above image segmentation method is inadequate for original root images with complex backgrounds, necessitating its continued improvement. The DeepLab series is currently the most effective semantic segmentation network tool, and it serves to further enhance the theoretical depth of the network model structure (Chen et al., 2014, 2017, 2018a,b). Among this series, DeepLabv3+ combines the advantages of encoder–decoder architecture and atrous spatial pyramid pooling (ASPP), which can capture rich contextual information from plant root images at various resolutions and segment clear root loci. Ayhan and Kwan (2020) proposed a DeepLabv3+ network improvement based on a weighting strategy, which is used to segment three vegetation cover types: trees, shrubs, and grasses. They showed that DeepLabv3+ is superior to the most advanced machine learning algorithms, i.e., SVM and random forests, in spatial information extraction and pixel segmentation.

In addition, in medical imaging (Guo et al., 2019), remote sensing images (Zhang et al., 2018), road scenes (Badrinarayanan et al., 2015), electrical equipment (Lin et al., 2019), and other high-pixel image segmentation applications, deep learning-based semantic segmentation networks are also used to improve the efficiency and throughput of traditional segmentation methods. Therefore, we think that a semantic segmentation method combined with the Minirhizotrons system and DeepLabv3+ network may offer a better approach to segmenting *in situ* plant root images, facilitating further research involving *in situ* root phenotypes.

Improving the efficiency and accuracy of *in situ* root image segmentation and exploring high-throughput automated methods for root phenotype analysis are of great significance for research pertaining to root phenotypes. This study proposes an improved and effective method for segmenting *in situ* root images from the Minirhizotron system, which was employed to obtain high-resolution images of cotton root systems. To improve the performance of segmentation of root images, a network design based on the encoder–decoder architecture of DeepLabv3+ was adopted, and the final upsampling part of the model was improved. This study evaluated the qualitative use of the network segmentation performance according to its accuracy, recall, and F1 score, and the segmentation results were compared with those of Rhizotrons manual segmentation and U-Net network, respectively.

## Materials and Methods

### Image Collection

The experiment was conducted in 2019 at the experimental station of Hebei Agricultural University in Baoding District (38.85°N, 115.30°E), Hebei Province, China, which is located in the Yellow River basin. The experimental site has a temperate climate.

#### Minirhizotron Installation

**FIGURE 1:**
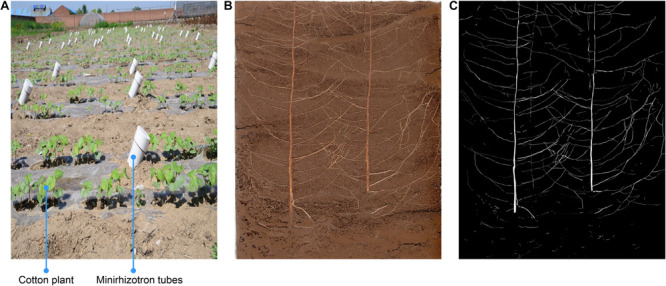
Minirhizotron installation and image annotation. **(A)** Minirhizotron tubes were installed at a 45° angle, parallel to the plant rows, and at a distance of 25 cm from the cotton plants in adjacent rows (halfway between rows), **(B)** Original image, **(C)** annotation image.

Eighteen Minirhizotron tubes were installed at a 45° angle, parallel to the plant rows, and at a distance of 25 cm from the cotton plants (halfway between rows). The tubes were made of plastic, and their bottoms were sealed. The total length of each tube was 200 cm, and the tubes reached a total depth of 150 cm (with 15–20 cm protruding from the soil). Light was restricted from the aboveground section of each tube by a black cover. The Minirhizotron tubes were installed during the winter of 2016 to ensure that the soil would be well distributed around the tubes and prevent roots from growing around the tubes. The device is shown in Figure 1A.

#### Root Image Acquisition

To measure root growth characteristics, images were recorded with a CI-600 scanner (CID Bio-Science, Inc., Camas, WA, United States). The scanner was connected to a laptop computer and was able to penetrate deep into the micro-root tubes and close to the inner wall to enable circular scanning. Images were captured at 20-cm intervals at nine positions along the tube with the aid of a connecting rod. The positions of the nine pictures are arranged in order from the deepest to the shallowest. The images were saved in the “bmp” file format.

#### Root Image Segmentation

In the method based on manual inputs, the images were analyzed with WinRHIZO Tron MF, which provided values for root length, projected root area, root surface area, and root volume based on users tracing the boundaries of each root using a mouse.

### Annotation

We conducted the screening and classification of a collection of cotton root images one by one. In the process, some incomplete or blurred images were removed, ultimately leaving 200 complete and clear *in situ* images of cotton roots. Among them, 20 selected root images were manually annotated for network training, and the remaining 180 were left as unexamined root samples that were used to evaluate network segmentation performance.

Image annotation was completed by experienced agronomy experts using the Adobe Photoshop CC (Adobe Inc., San Jose, CA, United States) lasso tool. All pixels considered to be roots were marked white and saved in a new layer, ultimately leaving the remaining pixels marked black (Figures 1B,C). Each root image was saved at 10,200 × 14,039 dpi resolution, and the annotation time for each image was approximately 4.5 h.

### Segmentation Model

DeepLabv3+ utilizes an encoder–decoder structure based on a fully convolutional neural network (FCN) (Chen et al., 2018b) and uses its previous model (DeepLabv3) as its own encoder and Xception as its backbone (Dai et al., 2017). In the root image segmentation task, the encoder is mainly used to extract the characteristics of the root morphology distribution. In the encoder portion, DeepLabv3+ does not blindly perform convolution-pooling operations like FCN. Instead, it uses the ASPP structure (Yang et al., 2018), which contains three parallel atrous convolutions with dilation rates of 6, 12, and 18 (Yu and Koltun, 2014), providing it a larger receptive field that can capture more root context information. Based on this approach, DeepLabv3+ also introduces the idea of depthwise separable convolution (Howard et al., 2017), which reduces the number of parameters while improving both running speed and classification performance. In order to fuse the multi-scale spatial information output by ASPP, feature concatenation is conducted using the concat approach, and channel compression is performed through a 1 × 1 convolution operation, which further reduces the network dimensionality and computation time. Finally, the encoder outputs a total root feature map that is 16 times smaller than the input image.

**FIGURE 2:**
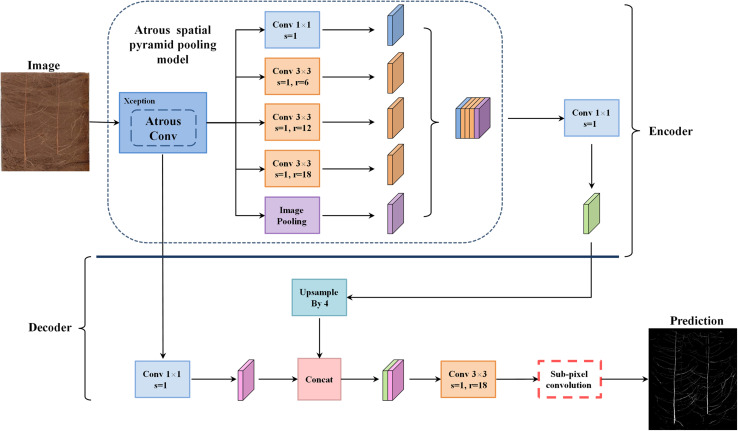
The proposed network architecture.

The main function of the decoder is to upsample the root feature map and to restore the details and boundary information for the root morphology distribution. In the decoder part, first, bilinear upsampling by a factor of 4 was used to change the encoding feature from output stride = 16 to output stride = 4, and then, the feature layer with the same spatial resolution (low-level) as in the encoder was extracted for skip connection. Then a 3 × 3 convolution kernel was used to fuse the combined total feature information, and finally, a 4-fold bilinear upsampling operation was performed on the fused feature to gradually restore the spatial size of the target root system and achieve semantic segmentation of the plant root morphology distribution. The improved model structure is shown in Figure 2.

### Model Improvement

The standard DeepLabv3+ network used the bilinear interpolation upsampling method to expand the size of the root fusion features in the final stage of the decoder output segmentation image (Chen et al., 2018b). As an interpolation algorithm in numerical analysis, bilinear interpolation is widely used in digital image processing. In deep learning tasks, bilinear interpolation is a common method for restoring image resolution (upsampling), which essentially performs two linear transformation operations. First, the *x* coordinate of the target pixel is linearly transformed, and the pixel values for the point *R*_1_ = (*x*,*y*_1_) and the point *R*_2_ = (*x*,*y*_2_) are, respectively, obtained. Then, another linear interpolation is performed on the pair of points *R*_1_,*R*_2_ to obtain the pixel value *R*_*P*_ at the point *P* = (*x*,*y*). This is summarized in Eqs. 1–3.

$$f\,\left(R_{1}\right)\approx \frac{x_{2}-x}{x_{2}-x_{1}}\,f\,\left(Q_{11}\right)+\frac{x-x_{1}}{x_{2}-x_{1}}\,f\,\left(Q_{11}\right)$$

(1) $$\,w\,h\,e\,r\,e\,R_{1}=\left(x,y_{1}\right)$$

$$f\,\left(R_{2}\right)\approx \frac{x_{2}-x}{x_{2}-x_{1}}\,f\,\left(Q_{12}\right)+\frac{x-x_{1}}{x_{2}-x_{1}}\,f\,\left(Q_{22}\right)$$

(2) $$\,w\,h\,e\,r\,e\,R_{2}=\left(x,y_{2}\right)$$

(3) $$f\,\left(P\right)\approx \frac{y-y_{1}}{y_{2}-y_{1}}\,f\,\left(R_{2}\right)+\frac{y_{2}-y}{y_{2}-y_{1}}\,f\,\left(R_{1}\right)$$

In Eqs. 1–3, points*Q*_11_ = (*x*_1_,*y*_1_),*Q*_12_ = (*x*_1_,*y*_2_),*Q*_21_ = (*x*_2_,*y*_1_),*Q*_22_ = (*x*_2_,*y*_2_) are known coordinates. Assuming that each pixel value along the coordinate axes satisfies the function *R*_α_ = *f*(*Q*_α_), then *R*_*P*_ is the calculated pixel value at point *P* = (*x*,*y*).

Although DeepLabv3+ uses bilinear interpolation upsampling to generate smooth segmented images, however, because the bilinear interpolation method only considers the influence of the gray value of the four direct neighboring points around the sample point to be tested, it does not consider the influence of the gray value change rate between each neighboring point. Thus, it has the properties of a low-pass filter, high-frequency components are degraded. Therefore, when the root features are restored to their original spatial size, the pixels at the edge of a root system will become blurry. To some extent, there are problems such as impaired image quality and low calculation accuracy that are caused by improper design of the interpolation function.

This article introduces the idea of the PixelShuffle algorithm (Shi et al., 2016) to perform pixel enhancement on the fusion features before the decoder output, replacing the second bilinear upsampling operation in the original DeepLabv3+ network, as shown in the red box in Figure 2. Sub-pixel convolution is an efficient upsampling method based on deep learning. In most cases, the convolution operation will extract target features and obtain low-resolution feature maps. However, when stride = 1/*r*(for *r* > 1), the length and width of the feature layer after the convolution operation will necessarily become larger, that is, the resolution will increase. This operation is called sub-pixel convolution (Aitken et al., 2017). Its initial definition is shown in Eq. 4:

(4) $$I^{S\,R}=f^{L}\,\left(I^{L\,R}\right)=P\,S\,\left(W_{L}*f^{L-1}\,\left(I^{L\,R}\right)+b_{L}\right)$$

here, *I*^*SR*^ refers to the finally restored high-resolution RGB image, *I*^*LR*^ refers to the low-resolution RGB image before restoration, *f*^*L*−1^ refers to a neural network with *L* - 1 layers, *W*_*L*_ and *b*_*L*_ are parameters of layer *L*, and *PS*(⋅) represents a shuffling operator that rearranges the elements of an *H*×*W*×(*C*⋅*r*^2^) tensor to a tensor of the shape r*H*×*rW*×C, where *r* is the upscaling factor. In this article, *r=2*.

**FIGURE 3:**
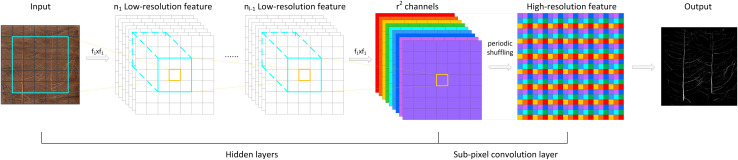
The PixelShuffle algorithm implementation flowchart.

The implementation of the PixelShuffle algorithm is summarized in Figure 3. The main concept of PixelShuffle focuses on the sub-pixel convolutional layer, which can convert a low-resolution input image of size *H* × *W* into a high-resolution image of *rH* × *rW* through sub-pixel convolution. In the whole process, there is no direct way to improve the resolution of target features through interpolation and other methods. Instead, first, a sub-pixel image is created from the original input image using fractional indices, and then, the feature map of *r*^2^ channels is generated by convolution (the feature map size is the same as the input low-resolution image), and finally, this high-resolution image is obtained by periodic shuffling. Such an approach can enable the network to learn an interpolation method suitable for the task in the previous convolution layer parameters.

Previous studies have shown that for the problem of image super-resolution reconstruction based on deep learning, an upsampling process based on sub-pixel convolution is effective in improving image quality (Zhao et al., 2019, 2020; Yu et al., 2020). Therefore, in the root pixel segmentation task, the introduction of sub-pixel convolution can improve the problem of pixel loss after bilinear upsampling in the standard DeepLabv3+, thereby improving the segmentation accuracy of the network for small root loci and further enhancing the robustness of the model.

### Network Training

We randomly selected 10 of the 20 root images with annotations to form the training and validation sets, respectively. As the original images used were too large for GPU memory allocation, we split the 10 root images and their corresponding annotations into 512 × 512 sub-images. In addition, zero padding was included for each image to ensure their dimensions would be divisible by 512.

The server environment for all computations used Ubuntu 16.04LTS and Python 3.6.7. The model was trained and tested under TensorFlow 1.13.1 and CUDA 10.2. The server was equipped with two NVIDIA GEFORCE RTX 2080Ti graphics cards for acceleration, and each graphics card had 10 GB of video memory.

We used three different sampling rates (6, 12, and 18) in the ASPP module of DeepLabv3+ to obtain multi-scale features of the target root system. A pixel-by-pixel cross-entropy loss function and an Adam optimizer (Kingma and Ba, 2015) were used to train the network. Adam is an efficient stochastic optimization method with low memory requirements. It can complete the initial stage of model training by adaptively adjusting the learning rate and quickly approaching the vicinity of the optimal solution, avoiding SGD to adopt a constant learning rate during training to update the weight. In order to improve the convergence speed of the network during training, after many tests, the initial learning rate, momentum β_1_, momentum β_2_, and epsilon were set to 7e-4, 0.9, 0.999, and 1e-8, respectively. In order to prevent the network from overfitting, the weight attenuation was set to 1e-6. Because a batch size that is too high will result in insufficient GPU memory, the batch size was set to 24, and 80 epochs were trained.

### Evaluation

In order to objectively and reasonably evaluate the effect of the network in the cotton root morphology segmentation task, this paper utilized three quantitative indicators, i.e., precision, recall, and F1-score:

(5) $$\text{Precision}=\frac{T\,P}{T\,P+F\,P}\times 100\%$$

(6) $$\text{Recall}=\frac{T\,P}{T\,P+F\,N}\times 100\%$$

(7) $$\text{F}\,1=2\times P\,r\,e\,c\,i\,s\,i\,o\,n\times \frac{R\,e\,c\,a\,l\,l}{P\,r\,e\,c\,i\,s\,i\,o\,n+R\,e\,c\,a\,l\,l}\times 100\%$$

In Eqs. 5, 6, TP is the number of pixels of the root distribution position that is correctly divided, FP is the number of background pixels that are incorrectly divided into the root distribution position, and FN is the number of root pixels that are incorrectly marked as the background. This paper uses precision to evaluate the global accuracy of the model, reflecting the proportion of true positive samples in the positive examples of root pixels determined by the classifier. Recall reflects the proportion of positive examples that are correctly determined to account for the total positive examples. F1 can be regarded as the weighted average of model accuracy and recall. For models with better segmentation performance, the coefficient is relatively high. In the model training process, the verification set was used to calculate the recall, precision, and F1 values of each epoch output in detail, and then, the model performance was evaluated using the test set that was not used in the training.

## Results

### Performance

After each epoch, accuracy and loss values were calculated on the training set to monitor its ability to generalize and avoid overfitting. After about 7 h and 8 min of training, the model accuracy and loss values tended to be flat after the 40th epoch, and a final accuracy of 0.9978 was obtained by the 80th epoch, with the loss ultimately stabilizing at approximately 0.0051 (Supplementary Figure S1). Table 1 shows the F1-score, recall, and precision of the proposed model in the verification stage. DeepLabv3+ ultimately achieved precision and recall values of 0.9702 and 0.9847, respectively, with the validation set, which means that the number of pixels in the model that mistake the soil background for the root is greater than the number of pixels that mistake the root for the soil background. Additionally, the overall standard F1-score of the model segmentation performance evaluation reached 0.9773, which demonstrates the high accuracy of our method.

**TABLE 1:** DeepLabv3+ segmentation performance evaluation on the validation set.

	**Accuracy**	**Precision**	**Recall**	**F1**	**Loss**
DeepLabv3+ (Improved)	0.9962	0.9702	0.9847	0.9773	0.0717

**FIGURE 4:**
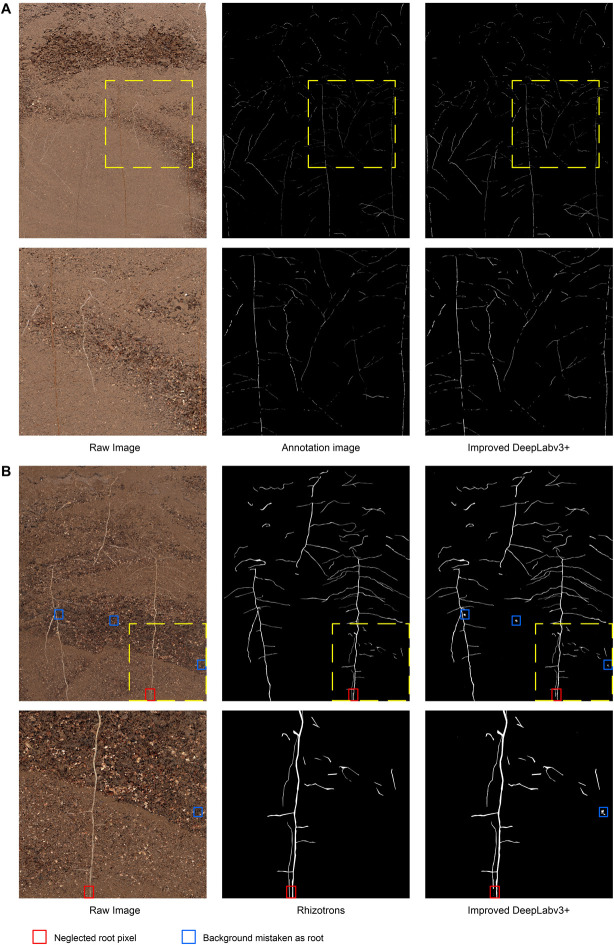
Examples and details of segmentation results. **(A)** The visual effect of the improved DeepLabv3+ in extracting root trajectories from *in situ* images of cotton roots, **(B)** Comparison of segmentation results of previously unanalyzed images. Red box, neglected root locus; Blue box, background mistaken for root.

Figure 4A illustrates the visual effect of the improved DeepLabv3+ in extracting root trajectories from *in situ* images of cotton roots. The predicted root pixels recovered most of the original root distribution path, reflecting a high similarity to the manual labeling conducted. The example image shown in Figure 4A was the most difficult to distinguish between soil particles and root pixels among the cotton root image data set obtained by Minirhizotrons. It has very small and irregular root trajectories and contains more obstructions by stones. Even human eyes have difficulty in quickly locating the root shape distribution in the image, which poses a huge challenge to the root segmentation task, but our improved DeepLabv3+ network can still more accurately mark the root distribution position with high-contrast contours. To see the results of manual tracking and the improved DeepLabv3+ prediction more clearly, we inserted an enlarged image at the same position in all six images in Figure 4 to show areas with noisy data.

### Untrained Root Image Prediction

In order to further evaluate the segmentation performance of this method for plant roots, we randomly selected 161 out of 180 root images that were unexamined, and, respectively, performed Rhizotrons manual segmentation and the improved DeepLabv3+ network segmentation. Figure 4B shows an example of images and predictions that have never previously been analyzed with the improved DeepLabv3+.

In the prediction results of 161 root images, no obvious performance degradation was observed, which is satisfactory in the segmentation results of most root shapes. However, there were still subtle errors, such as the number of brown root pixels (red box in Figure 4B) in the original image that were ignored by the network, and some of the highlighted soil stone particles (blue box in Figure 4B) under the network analysis were miscategorized as roots. The results show that the improved DeepLabv3+ has a good general ability to analyze root images that it has not been trained with. Additionally, thanks to the ability of Minirhizotrons system to some extent, it is able to obtain high-quality, high-consistency images.

**FIGURE 5:**
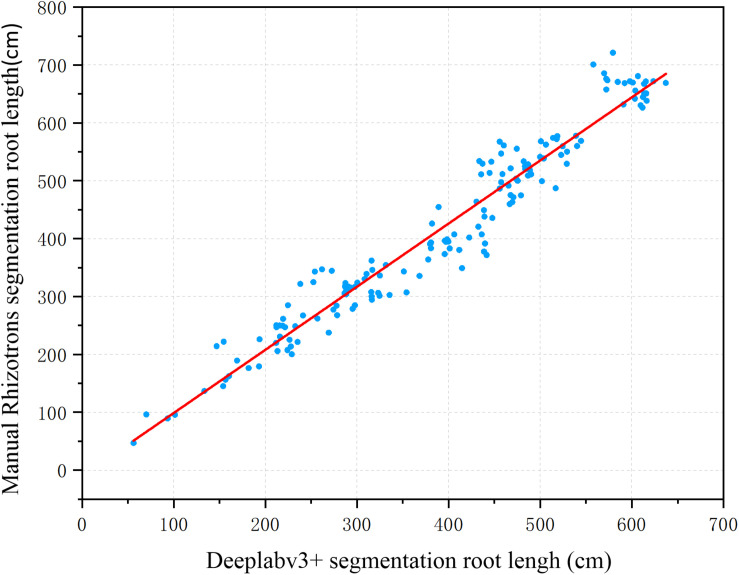
Root length estimation results (161 images). The two measurements have a Spearman rank correlation of 0.9667 and an *R*^2^ of 0.9449.

To further evaluate the segmentation performance of the proposed method for plant roots, the 161 root images segmented by the improved DeepLabv3+ and Rhizotrons were analyzed using WinRHIZO Tron MF to obtain four quantitative indicators of root length, surface area, volume, and average diameter for comparative evaluation (Table 2). The root length and average diameter were measured by WinRHIZO Tron MF, while the root surface area and volume were calculated from the root length and average diameter. In addition, a scatterplot and a fitting curve of the improved DeepLabv3+ and Rhizotrons manual segmentation of root length were also drawn, as shown in Figure 5. The Spearman rank correlation between the two measurements was 0.9667 (*p* < 10^–8^), with *r*^2^ = 0.9449. The comparison reveals that although the improved DeepLabv3+ and Rhizotrons manual segmentation have highly correlated root length results, root surface area, average diameter and total volume results still include obvious errors between the methods.

**TABLE 2:** Comparison of the improved DeepLabv3+ segmentation and manual segmentation results of 161 root images under WinRHIZO root analysis software.

**Phenotypic parameters**	**Length**	**Surf area**	**Avg diam**	**Root volume**
Spearman	0.9667	0.8624	0.0808	0.7955
*R*^2^	0.9449	0.7119	0.0062	0.5406

### Sub-Pixel Convolution Performance

**FIGURE 6:**
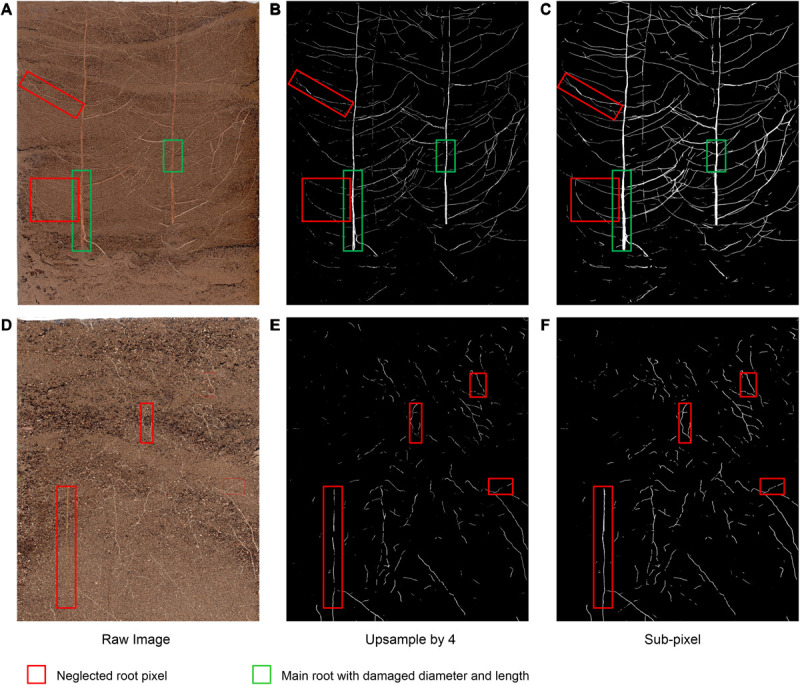
Example images of the comparison of the bilinear interpolation and subpixel convolution segmentation results of DeepLabv3+. **(A,D)** Raw image, **(B,E)** Original DeepLabv3+ segmentation results, **(C,F)** Improved DeepLabv3+ segmentation results.

To verify the effectiveness of the improved DeepLabv3+ method on the cotton root data set, we recorded the dice scores of different segmentation methods (i.e., standard DeepLabv3+, improved DeepLabv3+, and standard U-Net) on 161 root image segmentation task that has never been trained, as shown in Table 3. We compared the root segmentation results of the original network’s bilinear interpolation upsampling method and the sub-pixel convolution upsampling method, for example. As shown in Figure 6, two root images with different soil backgrounds were randomly selected from the 180 root images that had never been subjected to segmentation analysis.

**TABLE 3:** Performance comparison of different segmentation methods on the test set.

**Network**	**Testing dice score**
U-Net	0.5923
DeepLabv3+	0.6252
DeepLabv3(Improved)	0.6744

Under interference from two different soil background particles, the improved network can complete the segmentation of the original root system images with higher accuracy (Figures 6C,F),coincides with dice score. For Figure 6A, with higher contrast between root and soil, the traditional bilinear interpolation upsampling method functions similarly to a low-pass filter, such that some deep root features of the root system are degraded when returning to the original pixel value (red box in Figure 6B). Additionally, the loss of part of the main root diameter is visible to the naked eye (green box in Figure 6B). In situations such as the one shown in Figure 6D, where the contrast between the root system and the soil is low, the traditional bilinear interpolation upsampling method loses the continuity and brightness of the root system owing to interference from complex soil particles (red box in Figure 6E). The improved network can restore the multi-scale root features visible in the original pixels captured and maintain a high degree of restoration (Figure 6F). Thus, the improved DeepLabv3+ method can maintain the integrity of major roots and the continuity of the outline of the fine root edge, which can thus highlight the distribution characteristics of the fine root with higher contrast, thereby achieving improved segmentation results.

### Comparison With U-Net

To further compare the performance of other segmentation methods with the proposed network, we trained another network U-net that is widely used to perform segmentation (Ronneberger et al., 2015). U-net is a deep learning network composed of an encoder–decoder structure with jump connections. Its structure is more inclined to extract the global features of the input image and generate a new representation form based on the overall information. To ensure the consistency of the training process, we used the same 10 annotated *in situ* cotton root images as the input of the standard U-net and also conducted 80 epochs of training. The server training environment was the same as that used for DeepLabv3+.

Table 4 shows the precision, recall, and F1-score results of the improved DeepLabv3+ and the standard U-Net method in the verification stage; the precision, recall, and F1-score of the improved DeepLabv3+ were 0.9702, 0.9847, and 0.9773, respectively, while those of U-Net were 0.8413, 0.9489, and 0.8919, respectively. In addition, in the segmentation test of 161 root images, U-Net’s dice score (0.5923) is also lower than that of the improved DeepLabv3+ (0.6744), as shown in Table 3. Accordingly, our improved DeepLabv3+ outperformed the standard U-Net in all three metrics.

**TABLE 4:** Comparison of performance indicators between the improved DeepLabv3+ and the standard U-Net network segmentation in the verification stage.

	**U-Net**	**DeepLabv3+ (Improved)**
Precision	0.8413	0.9702
Recall	0.9489	0.9847
F_1_	0.8919	0.9773

**FIGURE 7:**
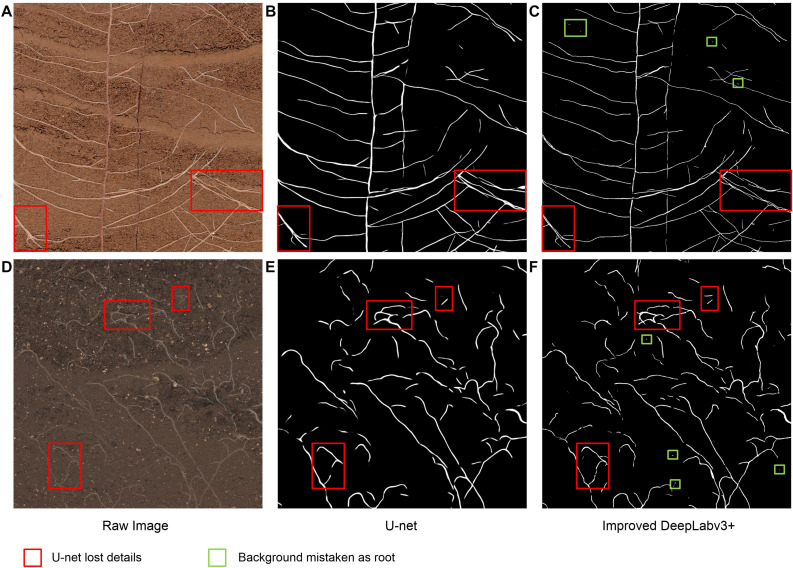
Example images used to compare the improved DeepLabv3+ and the standard U-Net segmentation performance. **(A,D)** Raw image, **(B,E)** Segmentation output from U-Net, **(C,F)** Segmentation output from the improved DeepLabV3+.

To compare the segmentation performance of the two CNN networks more clearly, part of the root structure was randomly intercepted from the test images that had never been seen, as shown in Figure 7. Compared with the improved DeepLabv3+, U-net’s root trajectory segmentation was too smooth and some of the visible details were lost (red box in Figures 7B,E). Additionally, the method we proposed was superior in terms of detail processing between root pixels and the prediction effect of the root edge contour. However, we also noticed some shortcomings; the improved DeepLabv3+, compared to U-net, had a tendency to mistake individual soil particles as root pixels (green box in Figures 7C,F).

## Discussion

Minirhizotrons visualizes root growth from pictures of soil profiles obtained by a camera or scanner through a glass or acrylic tube (Ohashi et al., 2019). It is considered a non-destructive method that enables monitoring of root growth across time and seasons (Kirkham et al., 1998). The high-resolution *in situ* root images collected by the Rhizotrons method generally segments roots and obtains root morphological indicators using WinRHIZO Tron MF software, which is a traditional manual segmentation method (Munoz-Romero et al., 2010). Further analysis of root morphological indicators can be used to obtain the dynamics of root phenotype changes, which is an advantage of the Rhizotrons method. However, this traditional manual segmentation method is greatly affected by human subjectivity, and the segmentation time is longer, approximately 2 to 3 h for an image, making it an inefficient method. Therefore, a high-efficiency and high-accuracy *in situ* root image segmentation method is needed to support *in situ* root phenotype research (Smith et al., 2020).

Improving image quality is the most important issue in *in situ* root system research. To improve the image quality, first, we embedded the micro-root tubes 12 months in advance to make the outer wall of the micro-root tubes close to the soil; second, the imaging equipment was protected to prevent scratches on the inner wall of the micro-root canal; third, before imaging, we brushed the inner wall of the root canal to reduce the influence of dust and determine whether there is water leakage.

In this study, a deep convolutional neural network based on DeepLabv3+ was implemented and tested for the purpose of automatic segmentation of root trajectories in soil. A micro-root window root system was used to obtain high-resolution *in situ* cotton root images, and WinRHIZO Tron MF was used in a comparative analysis of the segmentation performance of the proposed method. The root image segmentation quality obtained validates the effectiveness of the proposed segmentation method. Comparisons of the root image segmentation quality between the proposed methods and more established methods have revealed the efficiency of the proposed method.

To deeply analyze the segmentation performance of our proposed method, the results of the improved DeepLabv3+ network and Rhizotrons manual hand-drawn segmentation method were compared. Although the comparison results verify that the root lengths obtained with the improved DeepLabv3+ and Rhizotrons hand-segmentation were strongly correlated (Figure 5), there were still large errors in the root SurfArea, AvgDiam and RootVolume results (Table 2). The main explanation for the difference is that the automatic segmentation results of AvgDiam and the manual segmentation results of Rhizotrons have lower fitting performance (*r*^2^ = 0.0062). By observing the segmentation statistics of all root images, we find that the automatic segmentation result of AvgDiam always remains near 0.3mm, and does not change with roots of different diameters (Supplementary Table S1). In addition, the reason for the error of SurfArea and RootVolume is related to its calculation method:SurfArea and AvgDiam have a square relationship, while RootVolume and AvgDiam have a cubic relationship, So the error of AvgDiam will make the calculation error of SurfArea and RootVolume bigger. In addition, we believe that another factor that affects the disparity in root segmentation results is that the improved DeepLabv3+ can mistake a portion of soil particles that resemble roots to be root pixels (Figures 4, 7). Accordingly, our future work will focus on further improving the accuracy of the model, especially for the measurement of the average root diameter.

Although the standard U-Net method stitches features together in the channel dimension to obtain richer features, its upsampling results are still relatively smooth. For complex root images, it is easy to lose some details (Figure 7). Additionally, our improved DeepLabv3+ network introduces the PixelShuffle algorithm, which enables the network to learn an interpolation method that adapts to the root segmentation task, and then performs pixel enhancement on the fusion features before the decoder output. In the 161 image segmentation experiments that were never trained, our proposed model also achieved more accurate results (testing dice score of 0.6744). Therefore, the improved DeepLabv3+ achieves accurate segmentation of small branches in the root system with better performance in assessing new samples outside the training set. These colored boxes in Figures 4, 6, 7 are just examples to get a clearer contrast of some of the details.

The traditional Rhizotrons manual segmentation of each cotton root image takes an average of 4.5 h. In this paper, the 161 root images manually segmented by Rhizotrons required a total of more than 700 h, which is often not feasible in actual projects. The improved DeepLabv3+ model takes only 7 h to train from scratch. For *in situ* root image prediction, each image takes only 55 s, and 161 root image predictions take less than 3 h in total. Compared with Rhizotrons manual segmentation, end-to-end automatic segmentation saves a lot of time with a small error range, and we believe this will greatly promote the study of root morphology segmentation in soil.

Another issue worth discussing in this article is the number of training samples. The resolution of each *in situ* cotton root system image obtained by Minirhizotrons is as high as 10,200 × 14,039 dpi. We selected 20 of the 200 *in situ* images of cotton root system as quasi-training images. After many experiments, we found that if all 20 annotated root images are used for network training, the final model accuracy does not objectively improve, but the training time does double. As such, some of this limited dataset appears to indeed be redundant for the network learning. To ensure the diversity of the data, we finally randomly selected 10 of the 20 annotated images for network training. Another 10 of them were used as spare images. As the network input size of the improved DeepLabv3+ is 512 × 512, we generated 16,936 sub-images for network training and 892 sub-images for network verification by cropping portions of the original images. This method enabled DeepLabv3+ to successfully converge within 80 epochs of training. Accordingly, these 10 original root images are sufficient for the network. Moreover, too much training data is considered to not only be tedious but also cause models to be overfit.

The performance of CNN-based segmentation methods partially depends on annotation quality. Owing to the complexity of plant root systems, even experienced agronomists should be expected to introduce some errors when annotating thousands of roots. Obviously, reducing annotation errors as much as possible can somewhat improve the accuracy of target segmentation, because any choice of CNN depends on having correct annotation. Additionally, the process of annotating plant roots is also a very time-consuming task. In this study, the annotate of each root image required 300 min. Accordingly, looking for ways to improve annotation quality and save annotation time will be an important direction of future research.

Transfer learning is a method that uses existing knowledge to solve problems in different but related fields. The key goal is to complete knowledge transfer between related fields (Pan and Yang, 2010). In this study, the previous method of data labeling was time-consuming and cumbersome. However, the use of the DeepLabv3+ network structure proposed in this paper to train the root system data set must be started from scratch each time. Therefore, in future research, we intend to use the method of transfer learning to fine-tune the existing network using root images from different plants to further transfer our proposed method to root segmentation in other crops.

Image enhancement technology has proven to be a method that can improve the performance of CNN models (Perez and Wang, 2017). Further exploration of the application of image enhancement methods in data sets will also be a major direction of our future work. We have recently examined how generative adversarial network (GAN) based on deep learning can learn the characteristics of a class of data and generate similar data (Goodfellow et al., 2014). To solve the problem of randomly occurring apple diseases leading to insufficient image data sets, Tian et al. (2019) used CycleGAN to learn the characteristics of anthracnose apple images and transfer them into healthy apple images. Notably, the apple lesion images generated by CycleGAN have new backgrounds, textures, and shapes, which is very helpful for improving the performance and robustness of the models used for analysis. Such results show that we can also use GAN to extract the root soil characteristics of other crops into the cotton root image. This method is conducive to being integrated into further improvements of DeepLabv3+ ’s root segmentation performance and robustness under complex soil backgrounds.

## Conclusion

To improve the efficiency of traditional manual segmentation of plant root images, we have proposed and validated a trainable end-to-end deep learning method, a CNN approach implemented in DeepLabv3+, which can be used to segment plant roots efficiently. The CNN model proposed in this paper is based on the encoder–decoder architecture of DeepLabv3+ and improves the final upsampling operation of the network. Precision, recall, and F1-score were used to evaluate the network performance, achieving final verification set scores of 0.9702, 0.9847, and 0.9773, respectively. Additionally, WinRHIZO Tron MF was used to analyze data from 161 root images segmented by the improved DeepLabv3+ and the traditional Rhizotrons method, and four quantitative indexes, i.e., root length, surface area, volume, and average diameter, were obtained for comparative evaluation. The root length results of the improved DeepLabv3+ network had a higher Spearman rank correlation with the manual results, i.e., 0.9667 (*p* < 10^–8^) with *r*^2^ = 0.9449, compared with the Spearman rank correlation between the root length results of Rhizotrons and manual segmentation. Thus, the proposed method significantly improves the efficiency of root segmentation in soil, making it an efficient alternative to Rhizotrons manual segmentation. Additionally, compared with the U-Net network method, the improved DeepLabv3+ achieved a higher F1-score than U-Net (0.8919) and was observed to segment the *in situ* root images with higher pixel accuracy and quality.

## Data Availability Statement

The raw data supporting the conclusions of this article will be made available by the authors, without undue reservation.

## Author Contributions

CS, LL, and NW conceived the idea and proposed the method. LL, LZ, and NW contributed to the preparation of equipment and acquisition of data. CS and NW wrote the code and tested the method. CS, LZ, and LL validated the results. CS wrote the manuscript. LL, NW, LS, and JK revised the manuscript. All authors read and approved the final manuscript.

## Conflict of Interest

The authors declare that the research was conducted in the absence of any commercial or financial relationships that could be construed as a potential conflict of interest.
