# Supplementary figures and images for: High-Throughput in situ Root Image Segmentation Based on the Improved DeepLabv3+ Method
Source: Front Plant Sci. 2020 Oct 19;11:576791. doi: 10.3389/fpls.2020.576791 (PMC7604297; doi:10.3389/fpls.2020.576791)

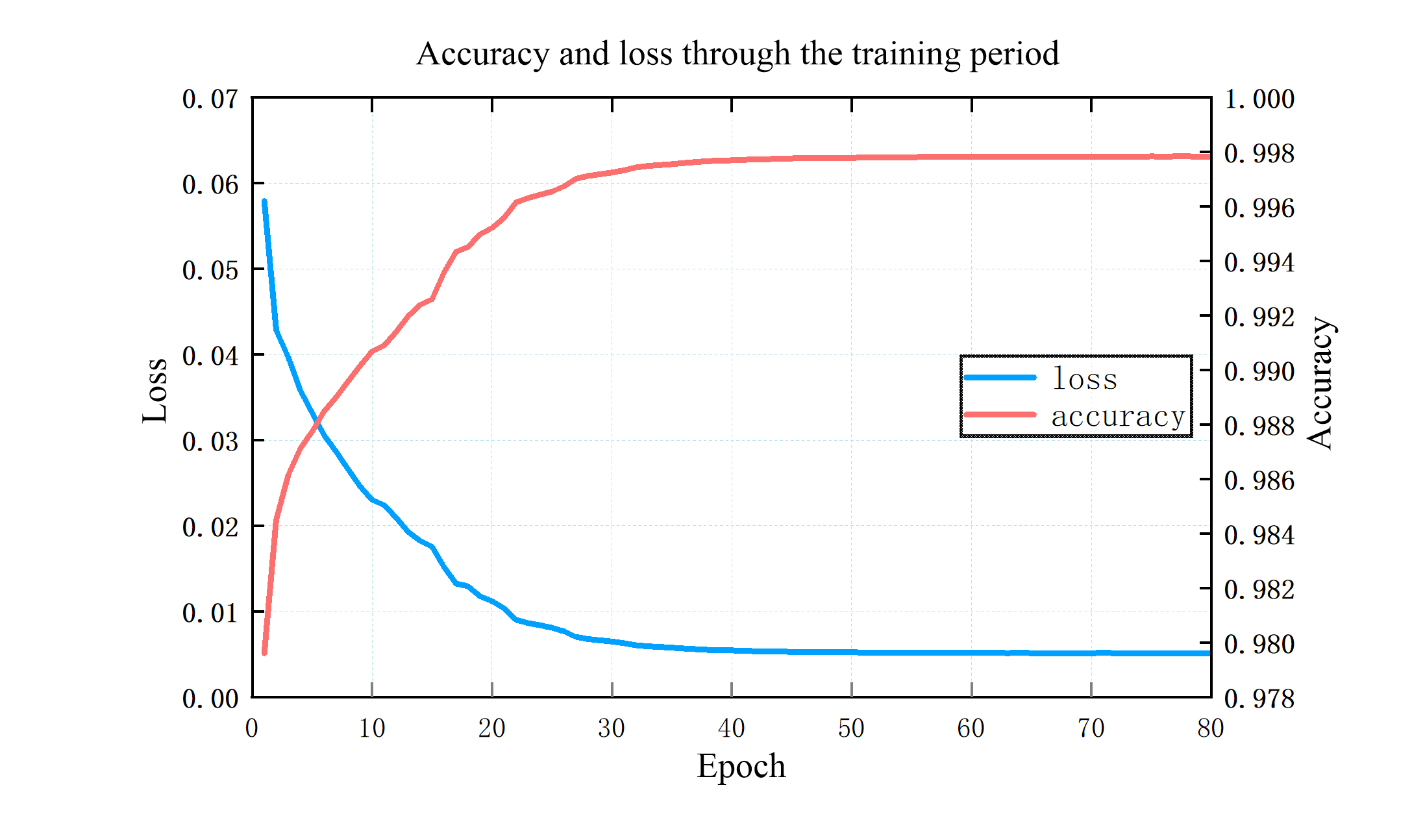

Supplement: Supplementary file 1 [file Image_1.TIF]
